# Infectious Disease Surveillance

**Published:** 2012-06

**Table:** Number of cases in Jan – Jun 2012*

Diseases	JAN	FEB	MAR	APR	MAY	JUN	Grand Total
AFP DISCARDED	55	59	17	50	64	62	307
AFP PENDING	5	8	16	6	8	2	45
AFP COMPATIBLE	0	0	0	0	0	0	0
AFP total	60	67	33	56	72	64	352
POLIO	0	0	0	0	0	0	0
AIDS CUMULATIVE			3455				3455
ANTHRAX	5	3	0	4	6	25	43
BRUCELLOSIS	890	821	887	868	1284	1416	6166
CCHF conf	0	0	1	8	26	28	63
CCHF Death	0	0	0	0	5	3	8
CCHF prob	2	9	4	17	75	75	182
CHOLERA	0	0	0	0	1	0	1
DIPHTHERIA-prob	14	13	8				35
HEPATITIS B	836	851	778	416	683	684	4248
HEPATITIS B&C	8	10	20	4	6	3	51
HEPATITIS B&D	0	0	0	0	0	0	0
HEPATITIS C	185	201	256	109	150	163	1064
HIV+ CUMULATIVE			24290				24290
HYDATIC CYST	49	47	67	4			167
LEISHMANIASIS CUTANEUS	2304	1778	1172	816	1362	671	8103
LEISHMANIASIS VISCERAL	5	5	3	1	13		27
LEPROSY	0	1	1	3	2	1	8
LEPTOSPIROSIS	0	1	1	0	5	3	10
MALARIA	12	9	17	32			70
MEASLES conf	2	3	17	30	53	16	121
MEASLES susp	268	233	198	300	560	410	1969
MENINGOCOCCAL MENINGITIS	2	2	1	2	1	0	8
NEONATAL TETANUS	0	0	0				0
OTHER MENINGITIS	107	97	96	139	161	89	689
PULMONARY TB			2079				2079
RABIES	0	1	0	0			1
RELAPSING FEVER	1	0	0	0	1	0	2
SYPHILIS	2	2	2				6
TETANUS	1	0	0				1
TUBERCULOSIS			2887				2887
TYPHOID & PARATYPHOID	44	22	40	14	46	50	216
WHOOPING COUGH- susp	119	111	46	3			279
AFP VDPV	0	0	0	0	0	0	0
GONOCOCCAL INFECTION	334	319	275				928

* Source: Iranian Center for Disease Control

